# Pediatric trauma deaths are predominated by severe head injuries during spring and summer

**DOI:** 10.1186/1757-7241-17-3

**Published:** 2009-01-22

**Authors:** Kjetil Søreide, Andreas J Krüger, Christian L Ellingsen, Kjell E Tjosevik

**Affiliations:** 1 Department of Surgery, Stavanger University Hospital, Stavanger, Norway; 2 Department of Surgical Sciences, University of Bergen, Bergen, Norway; 3 Acute Care Medicine Research Network, University of Stavanger, Stavanger, Norway; 4 Norwegian Air Ambulance, Drøbak, Norway; 5 Department of Pathology, Stavanger University Hospital, Stavanger, Norway; 6 Department of Acute Medicine, Stavanger University Hospital, Stavanger, Norway

## Abstract

**Background:**

Trauma is the most prevalent cause of death in the young. Insight into cause and time of fatal pediatric and adolescent trauma is important for planning trauma care and preventive measures. Our aim was to analyze cause, severity, mode and seasonal aspects of fatal pediatric trauma.

**Methods:**

Review of all consecutive autopsies for pediatric fatal trauma during a 10-year period within a defined population.

**Results:**

Of all pediatric trauma deaths (n = 36), 70% were males, with the gender increasing with age. Median age was 13 years (range 2–17). Blunt trauma predominated (by road traffic accidents) with most (n = 15; 42%) being "soft" victims, such as pedestrians/bicyclist and, 13 (36%) drivers or passengers in motor vehicles.

Penetrating trauma caused only 3 deaths. Prehospital deaths (58%) predominated. 15 children (all intubated) reached hospital alive and had severely deranged vital parameters: 8 were hypotensive (SBP < 90 mmHg), 13 were in respiratory distress, and 14 had GCS < 8 on arrival. Emergency procedures were initiated (i.e. neurosurgical decompression, abdominal surgery or pelvic fixation for hemorrhage) in 12 patients. Probability of survival (Ps) was < 33% in over 75% of the fatalities. A bimodal death pattern was evident; the initial peak by CNS injuries and exsanguinations, the latter peak by CNS alone. Most fatalities occurred during spring (53%) or summertime (25%).

**Conclusion:**

Fatal pediatric trauma occurs most frequently in boys during spring/summer, associated with severe head injuries and low probability of survival. Preventive measures appear mandated in order to reduce this mortality in this age group.

## Background

Worldwide, injuries and violence are the leading cause of death, in particular in the young. Road traffic accidents, self-inflicted injuries and interpersonal violence are leading causes of death in age-groups < 30 years in high-income countries, and falls represent a major disease burden [1]. The same pattern of mortality is noted in small children (1–4 years) with an increasing trend in high-income countries. In the United States (fiscal year 2003), there were 14,110 deaths from injury in children less than 18 years old reported to the National Center for Injury Prevention and Control. Of these, motor vehicle and traffic-related incidents were responsible for 63% of the deaths, followed distantly by homicide, suicide and drowning. The leading cause of nonfatal injuries was falls, and of the more than 8 million nonfatal injuries receiving medical attention, more than 151,000 required hospitalization [2].

Patterns of injury and death for the general trauma population are important for trauma systems, resource and management planning [3-6]. However, children and adolescents are recognized to have a particular set of injury patterns, severity, etiology and outcomes related to major trauma [4,7-9]. For one, they may participate in risk-taking behavior and activities that may be associated with major injuries, disability or even death [10,11]. While large epidemiological studies reflect regional or national trends and distributions [7,12-14], data derived from various registries may have limitations [15], and may miss valuable details from individual assessments of fatal trauma in the young. Also, there are few studies including post-mortem examination in population-based trauma assessment overall, and particularly in the younger victims. This is likely reflecting a general reluctance to performing autopsy as it has raised controversy by its value for trauma care evaluation [16-18]. The aim of the current study was to investigate the death of children and adolescents after trauma in a defined population. In particular, we wanted to investigate mechanisms, severity, and location of injury, as well as gender differences, and temporal and seasonal distribution of fatal trauma in the young.

## Methods and materials

### Study population

This study is based in part on a previous study on all trauma deaths in our region [3]. Aim of the current study was to investigate the deaths of children and adolescents resulting from major trauma forces and inflicted with severe anatomic injury. Thus, all traumatic deaths in victims aged < 18 years, and occurring in the Stavanger area during a 10-year period, beginning January 1^st^, 1996 and ending December 31^st^, 2005, and which underwent autopsy in our institution, were included. Patients were identified from a manual search of all autopsy records from this 10-year period. Excluded were drowning, hangings, poisonings, intoxications, and deaths exclusively caused by asphyxia with no anatomic injuries, and deaths from burn injuries.

Stavanger University Hospital (SUH) serves as the only primary trauma care facility for a mixed urban/rural population-based region of 290,000 inhabitants, and covers trauma for a wider population of approximately half a million. We have previously estimated a fatal trauma incidence of about 5–6 per 100 000 per year in those aged < 19-years in this region [3].

The prehospital emergency medicine service (EMS) system is based on paramedic-manned ambulances, in addition to an anesthesiologist-manned rapid-response car and helicopter emergency system (HEMS). The SUH has a designated trauma team, which responds within 5 minutes of activation, and is present in the trauma resuscitation room in the emergency department when the patient arrives [19]. Pediatric trauma is served by the general surgeon on-call, with pediatricians called in by priority, and intensive care initiated at a combined adult/pediatric surgical intensive care unit. Patients < 19 years of age-group represent about 20% of all trauma admissions, with the those aged 13 years or younger representing about half of the latter group (about 35 pediatric admissions/year fulfilling trauma registry criteria).

### Autopsies

Autopsies were performed at the Stavanger University Hospital, Department of Pathology. Post-mortem examinations were conducted by protocol [3]. Toxicology screens (blood and urine) were routinely performed in forensic autopsies. Postmortem radiological examinations were performed in select cases only [3]. Pre- and inhospital trauma deaths within the Stavanger County jurisdiction have a high autopsy rate due to a general agreement between the Stavanger Police Department and the forensic pathologists at the hospital. Due to national legislation, all prehospital trauma deaths should normally undergo forensic examination. Thus, we believe the current material of consecutive autopsies performed over a decade to serve as a reliable representative from a population-based Norwegian region.

### Data collection and definitions

Demographic data, injury pattern and severity, and physiological signs were obtained from prehospital trip charts, clinical charts, and forensic and medical autopsy records, whenever available. Systolic blood pressure (SBP), respiratory rate (RR), and Glasgow Coma Scale (GCS) were recorded on arrival in the emergency department, were applicable. To avoid missing values in physiological parameters, SBP, GCS, and RR were categorized on a five-point scale according to the Revised Trauma Score coded values [20]. Conservative scoring was achieved by not underscoring physiological signs if exact data were missing (i.e., if intubated, the patients' GCS were scored from pre-intubation information, or as GCS = 8 [RTS-code 2] if intubated and no other information on eye, verbal or motor response were available).

Injury severity scoring was performed by a registrar (K.S.) trained and certified in the methods by AAAM using the Abbreviated Injury Score (AIS-90, 1998 update), Revised Trauma Score (RTS), Injury Severity Score (ISS), New Injury Severity Score (NISS), and calculation of probability of survival (P_S_) for in-hospital deaths using the TRISS methodology [21-24].

Location of death was either prehospital or in-hospital. Temporal distribution was analyzed according to different time-intervals, as previously defined and reported [3,6]. Season of death was defined as Winter (December through February), Spring (March through May), Summer (June through August) and Fall (September through November).

The cause of death was defined as, either "central nervous system" (CNS), or "exsanguination", or "multiorgan failure syndrome" (MOFS) according to previously stated criteria [3,6].

### Statistical analysis

Statistical analysis was performed using SPSS version 13.0 (SPSS Inc., Chicago, USA). Comparison between continuous variables was performed with non-parametric Mann-Whitney U test. The Fischer's exact test was used for categorical data. All statistical tests were two-tailed, and significance level set at *P *< 0.05.

## Results

During the 10-year period, there were 36 autopsies performed for pediatric and adolescent deaths following trauma. Boys made up the majority of victims (n = 25; 70%), with demographics given in table 1. For those aged ≤13 years the number of girls almost equaled that of boys (8 girls vs. 11 boys), while the gender difference was more evident, although not statistically significant, in those aged 14–17 years (3 girls vs. 14 boys; P = 0.16).

**Table 1:** Characteristics of pediatric fatal trauma

		P-value*
Age, median (range)	13 (2–17)	0.82

Location of death		
Prehospital	21 (58%)	0.47
On-scene	19	
Transport	2	
Inhospital	15 (42%)	

Mechanism of injury		
Road traffic accidents	28 (78%)	0.084
Falls	4 (11%)	
Violence	4 (11%)	

ISS, median (IQR)	53 (33–75)	0.89

NISS, median (IQR)	66 (52–75)	0.59

Cause of death		
CNS	29 (81%)	0.99
Exanguination	7 (19%)	
MOF	0	

* for difference among genders

### Location and temporal distribution

Two of the 21 prehospital deaths succumbed during transport to hospital, and 15 (42%) reached the hospital alive before death. No significant difference in age (mean age of 11.6 yrs vs 10.8 yrs; p = 0.34) was noted between pre- and inhospital deaths, but statistically significant differences in ISS (mean ISS of 61.7 vs 39.6; p = 0.003), and NISS (mean NISS 65.7 vs 54.3;p = 0.01) were demonstrated.

**Figure 1:**
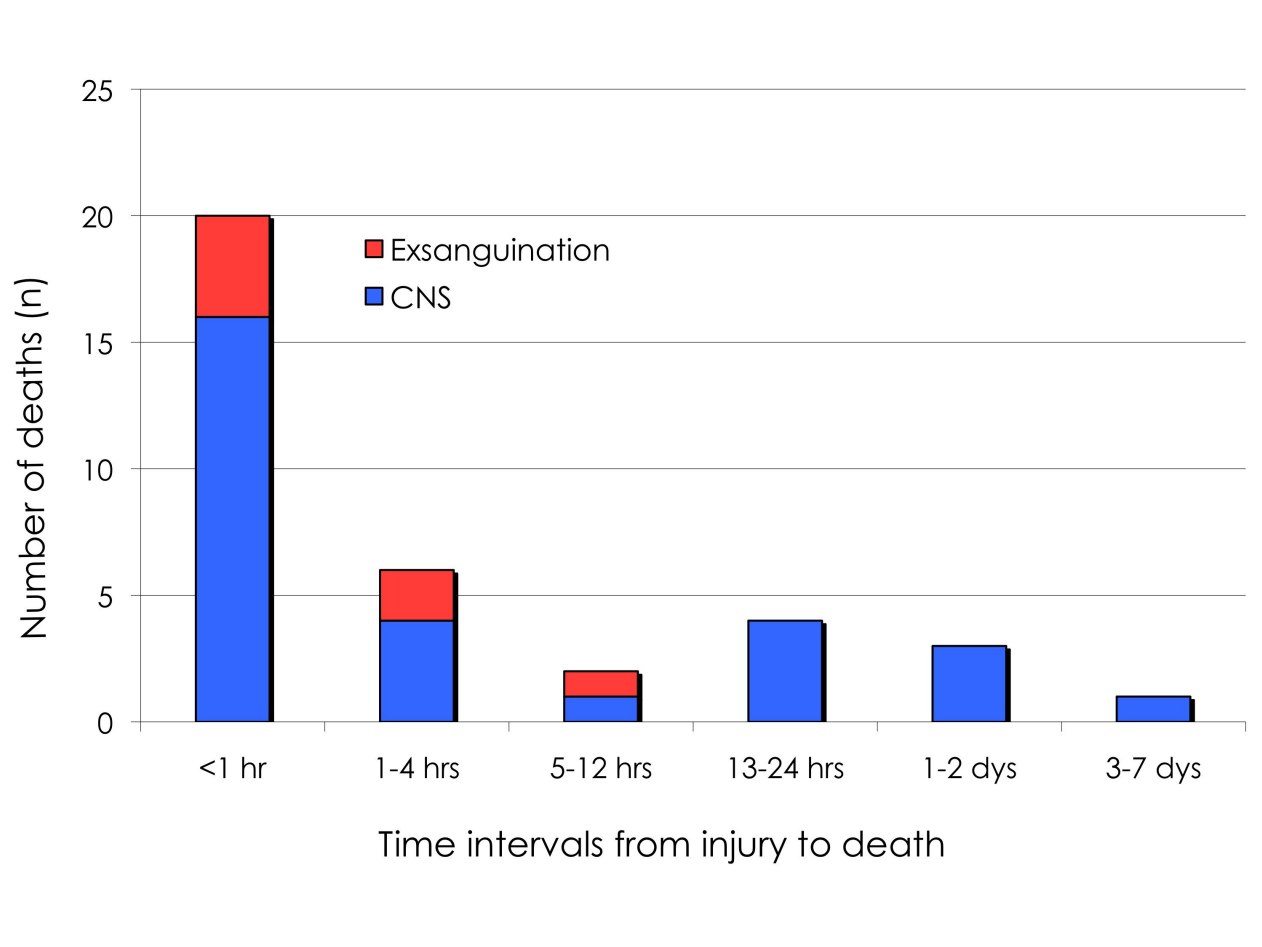
**Temporal distribution of pediatric trauma deaths**. Depicted is a bimodal temporal death distribution caused by early and late deaths from central nervous system (CNS) trauma.

A bimodal temporal death pattern was evident from the time from injury to death distribution (figure 1). Blunt mechanism was demonstrated in the majority (n = 33; 92%) of the victims, with no statistically significant differences between genders (P = 0.54), and penetrating trauma in only 3 children (8%), all of which were boys. Of the latter, all 3 were self-intentional handgun injuries and directed at the head, and only 1 reached the hospital alive. All 3 occurred during winter and fall. Road traffic accidents (RTA) caused the majority of blunt trauma, with most (n = 15; 42%) being "soft" victims, such as pedestrians/bicyclist and, 13 (36%) drivers or passengers in motor vehicles.

### Cause of death

CNS prevailed as the most frequent mode of death, and exsanguinations accounted for only 19% of deaths overall (2 of 11 girls; 5 of 25 boys). No child died of multiorgan failure.

### Toxicology screen

Of the 36 victims, 22 (61%) were screened for drug abuse, of which 17 (77%) tested negative for alcohol, benzodiazepines, cannabis, and amphetamines. 5 screens were positive (23% of all tested; or 14% of all victims); for alcohol in two girls and one boy, and two boys tested positive for cannabis. All positive tests were found in those ≥ 15 years of age, with only 6 patients ≤14 years of age tested.

### Seasonal trends

**Figure 2:**
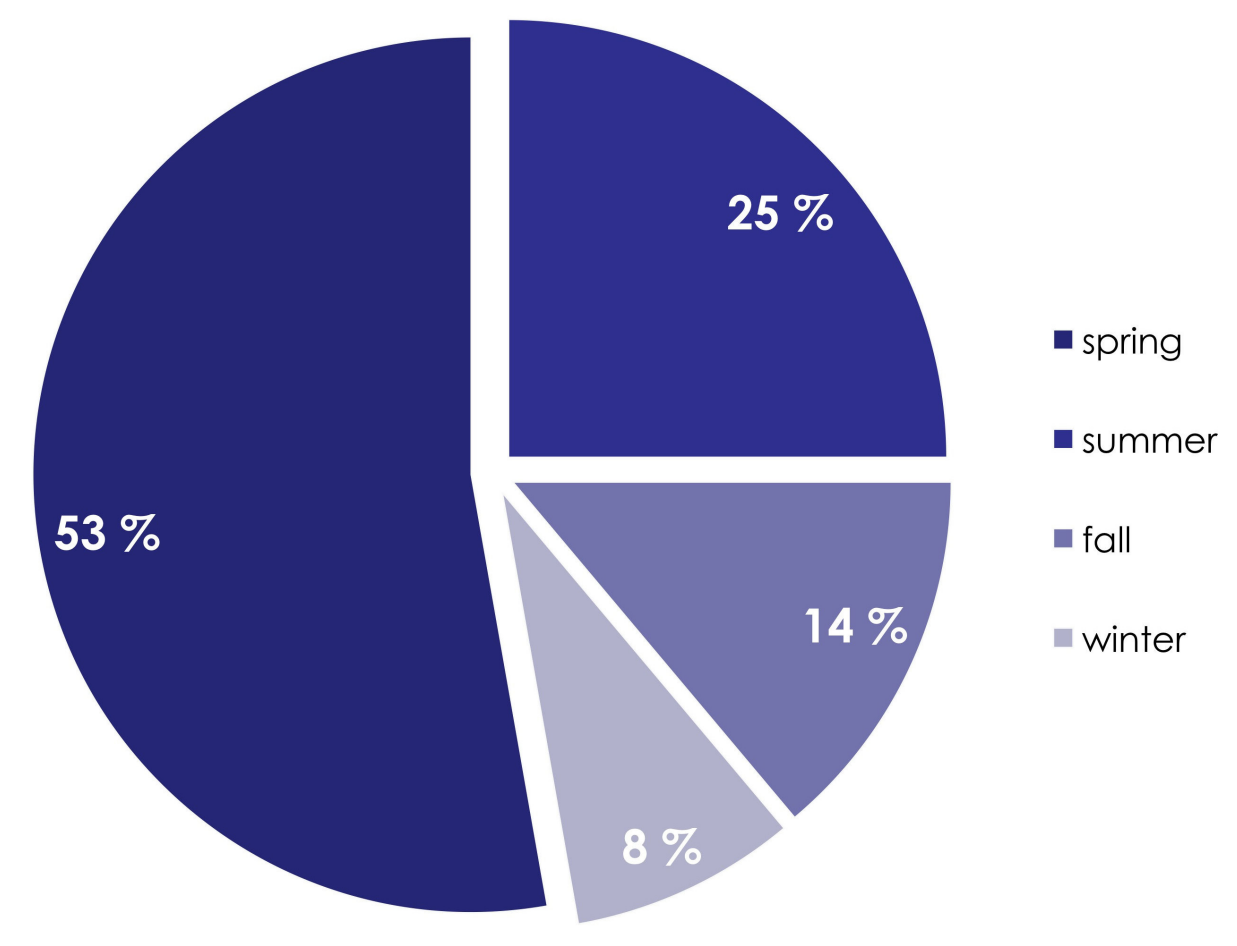
**Seasonal distribution of pediatric trauma deaths**. As depicted, the majority of deaths (78%) occur during spring and summertime.

The majority (75%) of fatal pediatric trauma occurred during spring and summertime (figure 2).

### Anatomical distribution and number of severe injuries

**Figure 3:**
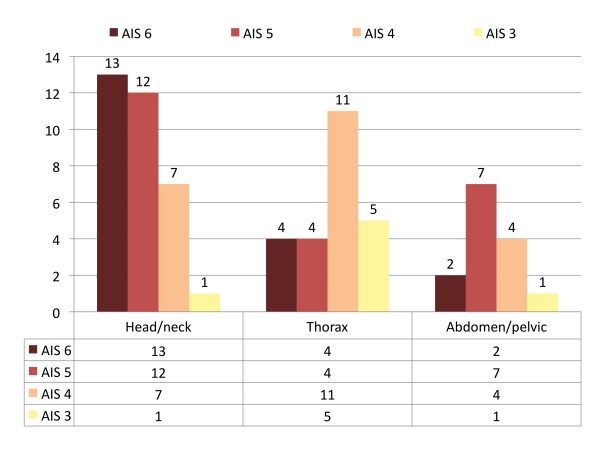
**Distribution of severe to fatal injuries in 36 children according to body region**.

A total number of 196 severe injuries (defined as any AIS ≥ 3) were documented in 36 children and adolescents, for median number of 5 severe injuries per child, with a range of 1–15. Distribution and number of severe to fatal injuries are given for the three most important body regions in figure 3. In 14 children an ISS of 75 was scored (and in 16 for NISS = 75).

### Vital signs

**Figure 4:**
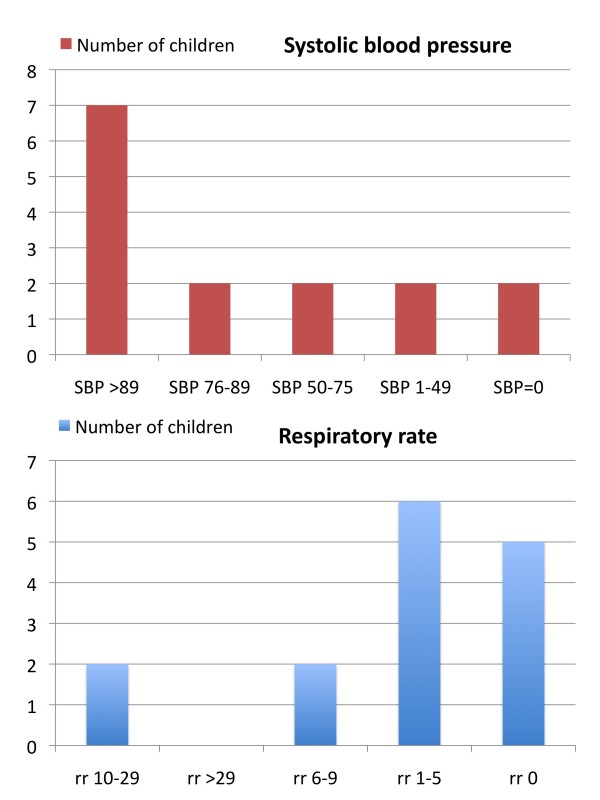
**Distribution of vital signs in 15 patients arriving to hospital**.

Of the 15 patients (6 girls, 9 boys) reaching hospital before death, 8 were hypotensive (SBP < 90 mmHg), 13 were in respiratory distress (RR < 10 or > 29/min), and 14 had GCS ≤8 on arrival, of which 11 had GCS = 3. Distribution of SBP and RR on arrival is given in figure 4. Seven of the children with GCS = 8 were also hypotensive. For the 15 patients arriving to hospital, 11 had head/neck injuries with AIS-score ≥ 5, six had thoracic injuries with AIS of ≥ 4, and 4 had abdominal injuries of AIS of ≥ 4.

The majority had very poor probability of survival (median Ps of 24%; IQR 5.8–33.5%), with only 1 having Ps > 50%. Median RTS was 2.49 (range 0–5.03). The emergency procedures performed for life-saving intent are listed in table 2.

**Table 2:** Emergency procedures performed in 15 children reaching hospital alive.

**Procedure**	**n**
Prehospital intubation	15

Thoracic drain insertion	4

Laparotomy	4

Neurosurgery	3

Pelvic fixation	1

## Discussion

Traumatic injury is the leading cause of death in children after infancy. The leading causes of childhood injury deaths are motor vehicle crashes, submersion injury, homicide, suicide, and fires [7].

This retrospective study of a consecutive autopsy series from a defined population in southwestern Norway shows fatal pediatric and adolescent trauma to consist of severely injured patients, with the majority of fatal injuries sustained to the head or to vital torso organs, with no, or only extremely poor, chances for survival for most victims. This is substantiated through the very high incidence of severe injuries, the location of these in the head/neck region, the number of very high ISS/NISS scores, and deranged physiology on arrival.

Limitations to the study, besides the retrospective design, is the exclusion of burns and other non-traumatic related causes of death (drowning, hanging, poisoning) which are often reported together with trauma-related deaths. However, we wanted to specifically focus on trauma-mechanisms resulting in anatomic injuries associated with a fatal outcome. Also, while this study was based on a very high autopsy rate in our region, some children with fatal trauma might have been missed when they became organ donors, as donors are often not undergoing additional autopsy (as the operative notes made after organ harvesting are usually regarded as a "partial autopsy report"). Thus, some children with isolated, severe head injuries may have missed the inclusion in this study. This would, however, have skewed the conclusion in an even stronger direction of our present finding of the head injury predominance.

As the numbers are small, statistical analysis should be interpreted cautiously, and statistics merely serve as an analytical adjunct to the clinical impressions in this study.

The role of head injuries is in line with other population-based investigations [9]. The importance of road traffic accidents is in line with previous reports from other areas [7,8,13,25]. Obviously it emphasizes the importance of preventive measures in this age group, as the potential for interventional or life-saving procedures for these injuries appear futile, although initiated in an attempt to save lives [26]. The pattern of injury mechanism concurs with that reported by WHO [1]. As such, the road traffic safety appears crucial for reducing the number of deaths in the young. The extrapolated estimate of about 700 pediatric trauma admissions fulfilling trauma registry criteria during the study period gives an estimated death incidence of 5,1% overall, or about 2% for inhospital deaths, which is higher than that reported for rural pediatric trauma in the US, but equals national statistics for inhospital deaths [9].

According to Norwegian national statistics (fiscal year of 2004) there are more than 2,550 deaths caused by external trauma in Norway each year. About 150 (6%) are reported in those aged < 19 years [14]. Male deaths predominate (1.6 times) over that of female deaths, with the largest gender difference in those aged 15–19 years (male: female-ratio of 2.7), and an near-equal distribution in those aged ≤14 years (boys: girls ratio of 1.2) [14]. The gender distribution is in accordance with the results obtained in this study.

However, in a previous report we showed that the age group < 19 years represent short of 20% of all trauma-related deaths within a defined population [3], thus questioning the validity of the Norwegian national Cause of Death statistics as these are based, and rely, on the accurate reporting and coding practices among regions and hospitals. Autopsy practice may vary significantly among regions, and thus trauma-related deaths may be underreported. This should deserve further attention, and mandates the need of a national trauma registry, which is currently called for in Norway.

As injuries are not completely random events, factors associated with injuries allow identification of high-risk populations and targets for intervention. Injury research includes development of conceptual models to include pre-injury, event, and post-event features that can be modified to prevent or limit injuries. Successful prevention strategies often include multifaceted approaches such as education, incentives for safe human behavior, legislation/law enforcement, and environmental changes [7]. Preventive programs must weigh both societal and economic values and costs. Careful evaluation for effectiveness of injury prevention programs to decrease or limit injury continues to be a challenge. Focus on injury prevention for penetrating trauma (i.e. handguns and firearms) appears less important in Norwegian pediatric fatal trauma, compared to US reports [2,12,27,28].

Somewhat surprising was the high number of deaths occurring during summer/spring-time, outnumbering deaths during autumn and wintertime. In southwestern Norway, daylight is reduced during the latter period (October through April) with dusk typically setting in when children are walking home from school, which has led to safety programs issued in media and schools with focus on traffic safety for children. Less focus has been issued on the same safety issues during summertime, when daylight and dusk periods are extended (almost until midnight for some periods) – however, more children may be active and out on the streets for a longer time during this time of year, and thus increasing the "time under exposure", i.e. for road traffic injuries. These observations are speculative at this stage, but should deserve further attention in future studies on causes and preventive strategies for pediatric trauma.

## Abbreviations

(Ps): Probability of survival; (CNS): Central nervous system; (MOFS): Multiorgan failure syndrome"; (HEMS): helicopter emergency system; (SBP): Systolic blood pressure; (RR): respiratory rate; and (GCS): Glasgow Coma Scale; (RTS): Revised Trauma Score; (ISS): Injury Severity Score; (NISS): New Injury Severity Score.

## Competing interests

The authors declare that they have no competing interests.

## Authors' contributions

KS conceived and designed the study. KS, AJK, CLE and KET collected the data. KS performed the data analysis. KS drafted the manuscript. All authors interpreted data and critically revised the manuscript. All authors have read and approved the final manuscript.
